# The effect of training interventions of stigma associated with mental illness on family caregivers: a quasi-experimental study

**DOI:** 10.1186/s12991-018-0218-y

**Published:** 2018-11-15

**Authors:** Farshid Shamsaei, Fatemeh Nazari, Efat Sadeghian

**Affiliations:** 1 Behavioural Disorders and Substance Abuse Research Centre, Hamadan University of Medical Sciences, Hamadan, Iran; 2 Department of Nursing and Midwifery, Hamadan University of Medical Sciences, Hamadan, Iran; 3 Chronic Diseases (Home Care) Research Centre, School of Nursing and Midwifery, Hamadan University of Medical Sciences, Hamadan, Iran

**Keywords:** Family caregivers, Mental illness, Stigma, Training

## Abstract

**Background:**

Stigma is one of the most destructive features of mental illnesses that may affect the family caregivers. This study aimed to analyze the effect of training interventions of stigma on family caregivers of the mental illness patients.

**Materials and methods:**

This quasi-experimental pre- and post-test study was performed on a single group of 43 family caregivers of mental illness patients in Hamadan Psychiatric Hospital, Iran, in 2015. The samples were taken through convenience sampling method and the data collection tool was a stigma questionnaire made by the researchers. The questionnaires were filled by the participants within pre-intervention and 1-month post-intervention. All the data were analyzed by SPSS version 16, and the mean and standard deviation by paired *t* test and Wilcoxon test.

**Results:**

Findings of this study demonstrated that women included 60% of the family caregivers. The average age of caregivers and the duration of caregiving were 41.67 ± 11.62 years and 66.28 ± 7.99 months, respectively. The mean and standard deviation for pre-intervention stigma score were 82.47 ± 12.23 indicating that the family caregivers suffered from some problems arisen from living with mental patients. They include not getting married, unable to find a job, embarrassment, humiliation by others, disgrace, and shame. Our results revealed that the mean and standard deviation of stigma score decreased to 29.28 ± 7.52 after training, and this difference was statistically significant (P < 0.001).

**Conclusions:**

According to the results of present study, training interventions reduce the issues caused by stigma and help the family members of mental patients to face and cope with the problem.

## Introduction

Family caregivers play the most prominent role in care-giving for mental illnesses patients, and there is a growing body of literature on the family burden and stigma, lack of caregiver support, and equivocal success, with interventions aiming at alleviating the care-giving burden [1, 2].

Family caregivers have to bear the negative effects caused by prejudice and stigmatization in addition to support of the patients both emotionally and physically. Stigma is one of the most destructive features of mental illnesses that may affect the family caregivers, patients’ families, and the patients themselves [3, 4]. In other words, many authorities in the field of psychological health believe that the most important obstacle to the mental patients treatment is “mental illness stigma” rather than the medication shortage, specialists, or facilities [5].

The stigma arisen from taking care of mental patients leads the prejudice, losing social status, preventive behavior strategies such as withdrawal, decreased life quality, disease intensity, drug abuse, failing to take the medications and to pursue the treatments, and confusion in the family [6]. Link and Phelan [6] announced that stigma includes five elements of labeling, stereotyping, cognitive isolation, emotional reactions, and prejudice so that a person in the society is labeled for any special characteristic which is different from the formalities of society and placed in minority [7, 8].

The stigma results the feeling of embarrassment in many family caregivers of mental patients. It should be noted that a low percentage of these members undergoes education and sufficient information considering the mental illnesses, signs and symptoms, correct approaches for facing the patients, and stereotyping [9–11].

Limited and mistaken information about psychological health and tendency for hiding the family member illness in family caregivers leads a remarkable augmentation in being stigmatized [12]. One of the effective approaches in reducing the stigma and omitting the negative attitude of society to these patients is to help the family caregivers understand the illness, encourage to accept pharmaceutical therapy, identify the early symptoms of relapse, and assure the rapid omission of disease attacks. The mentioned practices may result in better recovery of the patient and reduced social and personal disabilities. In addition, they might lead the family caregivers to better play their supportive and therapeutic roles [13].

In spite of various research projects on stigma reduction programs, few studies have examined how to overcome stigma toward family caregivers of mental illness patients [14, 15]. It seems that the stigma reduction strategies vary according to the contextual factors including politics, socioeconomic status, culture, religion and media. Iran is a Middle-East Islamic country with an approximately 79-million population [15] in which religious culture is dominant. In Iran, families play the key role in taking care of the mental illness patients, and social variables as well as the misbeliefs of people pose them some problems such as stigma. Therefore, this study was performed to analyze the effect of training stigma interventions associated with mental illness on family caregivers.

## Materials and methods

This quasi-experimental pre- and post-test study was conducted on a group of patients in Hamadan Psychiatric Hospital, Iran, in 2015. The sample included 43 mental patients family members who had the most prominent role in taking care of the mental patients and were selected through convenience sampling method. Sample size was measured according to a similar study [16] with reliability level of 95% and statistical power of 80% using the following equation:

$$ n{ = }\frac{{\left( {{\text{Z1} - }\alpha / 2  + {\text{ Z}1 - /2}} \right)^{ 2} {\text{S}}^{ 2} }}{{d^{ 2} }} = \frac{{\left( { 1. 9 6 + {0} . 8 4} \right)^{ 2} \left( {4.64} \right)^{ 2} }}{{ ( 2 )^{ 2} }}= {43} $$

The study inclusion criteria: (1) mental illness of one of the family members (e.g., schizophrenia, schizoaffective, bipolar disorders type I, major depressive disorder, etc.) diagnosed by the psychiatrist based on the diagnostic criteria of the 5th edition of the Diagnostic and Statistical Manual of Mental Disorders (DSM-5), (2) at least 1-year experience in taking care of the mental patient, (3) the patient is an adult, and 4) lack of mental retardation, chronic diseases, and drug addiction history in the family. The exclusion criteria included absence in more than two sessions of the trainings during the study and incidence of unpredicted stressors in the family.

To design the questionnaire as the data collection means, a full literature review was performed using different databases including *PsycINFO*, SID, Prequest, Up-to-date, Scopus, Pub med and Ovid. Therefore, the stigma evaluation questionnaire was designed for the family caregivers of the patients with chronic mental disorders. Afterwards, face and content validity in both quantitative and qualitative aspects, construct validity in addition to the internal consistency were all assessed. The primary tool consisting of 38 items was analyzed regarding the face and content validity based on the qualitative and quantitative features (CVR and CVI). In this stage, some items were omitted and merged, reducing to 33 items. The construct validity was examined through exploratory *factor analysis* and sample size of 356 which led the final remaining 30 items. The results of Cronbach’s alpha (0.83) and retest (0.87) were indicative of a high internal consistency and reliability of the tool.

Each item was answered as a Likert scale with five choices (i.e., never, rarely, sometimes, often, and always). The scores of zero, one, two, three, and four were attributed to the answers never, rarely, sometimes, often, and always, respectively. Therefore, the minimum score for the questionnaire was determined as zero and the maximum 120. Overall, the score ranges of 0–29, 30–59, 60–89, and 90–120 were interpreted as weak, moderate, severe, and strongly severe stigma, respectively, categorized into four groups of 25%.

### Intervention

Mental Illness Stigma Reduction Programs: a large number of programs and initiatives have attempted to reduce mental illness stigma. They can be roughly divided into two categories: training interventions that involve in-person communication between an educator/speaker and a small moderate-sized group, and mass media campaigns and broad multifaceted interventions. Some initiatives include both of these components [16, 17]. Training interventions typically involve an educational component in which information about the causes of mental illness, mental health treatment, and the experiences of people with mental health problems are provided to counteract the stereotypes and prejudice, and promote attitudes affirmation to the people with mental illness [18].

In this study, we used training interventions that involve in-person communication between an educator/speaker and a small group.

The content of the intervention sessions:

1. Providing information about the research and family’s experience of stigma.
  The purpose of this session was to meet the family caregivers, to provide them with information about the research objective and to determine the time and place of the education to be given. In addition, in this session, basics of psychological health were explained and the participants were asked to share and discuss their experiences about stigma if any.
2. Providing information about the mental illness.
  Aim of the second session was an introduction to mental illnesses and their reasons, treatments, and taking care of the mental patients. These aims were achieved by giving presentations and delivering pamphlets.
3. Providing information about roles of family in taking care of mental illness.
  The third session was held to clarify the importance and roles of the family in treatment and care for the mental patients. Therefore, the roles of family members in treatment and interactions with the patients were discussed in groups.
4. How to know stigma and teach skills for coping with stigma.
  In the fourth session, the purpose of stigma analysis is the effective factors on causing it, effects of accepting the stigma on treatment protocols, problems due to the stigma in families, confronting the stigma, and beliefs in the mental illnesses.

When participants were selected, they were divided into 9 groups (Each consisting of 4–5 people). Pretest was done before intervention. The education program included four sessions that lasted 60–75 min. We used a different day in the same week for each group. Sessions started with an evaluation of the past session. All participants completed the education program.

Education program was presented by a psychiatric nurse in cooperation with an associate professor of nursing. Post-test occurred 1 week after intervention.

### Statistical analysis

Data were analyzed using SPSS 16 packet program. The descriptive analysis included absolute and relative frequency distribution, mean, and standard deviation. Moreover, comparison of the mean scores, paired *t* test, and Wilcoxon test were utilized for the analytical statistics. The significance was set at *α *= 0.05.

## Results

According to the findings of our study, the average age of participants was 41.2 years, mostly consisting of women (60.5%). The average duration of taking care of the patients was 66.3 months and average duration of taking care during a week was 71 h (Table 1). The most common disorders among the patients were bipolar disorders type I (44.2%), obsessive–compulsive disorders 26.4%, Major depressive disorder 21% and schizophrenia 9.3%.

**Table 1:** Socio-demographic characteristics of caregivers

Characteristics	*N*	%
Sex		
Male	26	60.5
Female	17	39.5
Ages (years)		
> 30	6	13.9
30–39	12	27.9
40–49	21	48.8
≤ 50	4	9.3
Marital status		
Single	11	25.6
Married	27	62.8
Divorced	3	7
Widowed	2	4.6
Educational level		
Primary school	9	20.9
High school	27	62.8
University	7	16.3
Employment status		
Employed	10	23.2
Unemployed	3	7
Retired	2	4.6
Business	11	23.6
Agricultural worker	9	20.9
Housework	8	18.6
Relationship with patient		
Spouses	10	28.3
Parents	21	40.6
Children	7	12.9
Siblings	5	18.2

The mean stigma score pre-intervention was 82.47 ± 12.23, which declined to 29.28 ± 7 post-intervention. The difference of stigma questionnaire score between two pre- and post-intervention times was statistically significant (*P* < 0.001) (Table 2). The latter finding demonstrates that short-term training programs can also reduce the stigma in family caregivers of mental patients.

**Table 2:** Comparison of mean scores of stigma before and after intervention

Stigma score	Before intervention *N* (%)	After intervention *N* (%)	*P*
Mild	0	24 (55.8)	< 0.001*
Moderate	1 (2.3)	19 (44.2)	
Severe	30 (69.8)	0	
Strongly severe	12 (27.9)	0	
Mean ± SD	82.47 ± 12.23	29.28 ± 7.52	< 0.001**

* Wilcoxon signed rank test

** Paired sample *t* test

## Discussion

Results of the current study indicate that the mean stigma score is high in family caregivers of the mental illness patients before the training intervention. In other words, stigma is one of the problems arisen from caregiving and living with a mental patient as mentioned in the literature [19–22].

The present study aimed to investigate the effects of training on encountering the stigma in family caregivers of the mental illness patients. Our results showed that education could be effective on reducing the stigma score among the family caregivers.

The studies performed by Uchino and Cuhadar demonstrated that training could diminish the stigma in family caregivers of the patients with schizophrenia and mood disorder, which are compliant with the findings of this study [23, 24]. On the other hand, Kiropoulos et al. showed in Australia that training had no influence on stigma, which is not in line with our results [25].

The inconsistency between the current study and the study conducted by Kiropoulos et al. might be due to two major reasons [25]. The first reason is that training in the present study was face-to-face and direct contact with the caregivers. Corrigan et al. also emphasizes the positive and remarkable impact of direct training [5], while Kiropoulos et al. performed their training intervention via internet. Furthermore, their study population included several different cultures, such as Greeks, English-speaking people, and Italians. However, all the participants of the present study were familiar with Persian language and almost enjoy the similar culture [25].

Moreover, results of the studies performed by Bernhard et al. and Yang et al. indicated that training, knowledge, and attitude of the caregivers may improve the mental illness [11, 16]. In addition, Cuhadar et al. and Cook et al. also reached the conclusion that the hiding rate by the caregivers significantly declined post-intervention [24, 26].

Moreover, the findings of current study revealed that the preventive behaviors of the caregivers diminished, and their social interactions increased after training, as they are compliant with the results of Uchino et al. [23, 24, 26].

It was observed in the present research that the parents of mental patients did not blame themselves anymore after the training sessions, and they have mainly solved their ideas toward this problem. Accordingly, Yin et al. and Cuhadar and Cam mentioned in their studies that parents considerably blamed themselves less after the trainings [20, 24].

One of the limitations of the current study was the short period of training program, as it seems that long-term trainings and continuous follow-ups improve the efficacy of interventions. Moreover, the sample size could be considered as another limitation, and a more comprehensive study with larger sample size will enhance the generalizability of the results. In addition, applying a research-made questionnaire was also among the limitations of this study. Another limitation is related to sampling method. The data were collected by convincing sampling. This method may represent the views of a specific group rather than the entire population.

Despite the aforementioned limitations, findings of this study provide crucial empirical evidence regarding the effects of stigma confrontation training on family caregivers of people with mental illness in the Iran.

## Conclusions

This study demonstrated that stigma is one of the problems and challenges of mental patients’ family caregivers, and insufficient knowledge regarding the stigma phenomenon might exacerbate the problem. Therefore, the current research provided an evidence for the short-term efficacy of the training program in improving stigma-related knowledge of family caregivers of mental illness people.

## Abbreviations

CVI: content validity index
CVR: content validity ratio
DSM 5: diagnostic and statistical manual of mental disorders 5th edition
*PsycINFO*: *psychological information*
Pub med: pub medline
SID: scientific information database
SPSS: statistical package for the social sciences
